# Supplementary material for: COZOID: contact zone identifier for visual analysis of protein-protein interactions
Source: BMC Bioinformatics. 2018 Apr 6;19:125. doi: 10.1186/s12859-018-2113-6 (PMC5889581; doi:10.1186/s12859-018-2113-6)
Supplement: Supplementary file 2 — Software build. Executable binary file of the software tool. (ZIP 118,784 kb) [file 12859_2018_2113_MOESM2_ESM.zip › cozoid/platform/docs/testng-6.5.1-javadoc.zip!/org/testng/annotations/AfterSuite.html]

AfterSuite (TestNG)


---


|  |  |  |
| --- | --- | --- |
| **Overview** \| **Package** \| **Class** \| **Tree** \| **Deprecated** \| **Index** \| **Help** | |  |
| **PREV CLASS**   **NEXT CLASS** | **FRAMES**    **NO FRAMES**     **All Classes** |  |
| SUMMARY: REQUIRED \| OPTIONAL | DETAIL: ELEMENT |  |


---


## org.testng.annotations Annotation Type AfterSuite

---

``` @Retention(value=RUNTIME) @Target(value=METHOD) public @interface AfterSuite ```

---

| **Optional Element Summary** | |
| --- | --- |
| `boolean` | `alwaysRun`             For before methods (beforeSuite, beforeTest, beforeTestClass and beforeTestMethod, but not beforeGroups): If set to true, this configuration method will be run regardless of what groups it belongs to. |
| `java.lang.String[]` | `dependsOnGroups`             The list of groups this method depends on. |
| `java.lang.String[]` | `dependsOnMethods`             The list of methods this method depends on. |
| `java.lang.String` | `description`             The description for this method. |
| `boolean` | `enabled`             Whether methods on this class/method are enabled. |
| `java.lang.String[]` | `groups`             The list of groups this class/method belongs to. |
| `boolean` | `inheritGroups`             If true, this @Configuration method will belong to groups specified in the @Test annotation on the class (if any). |
| `long` | `timeOut`             The maximum number of milliseconds this method should take. |

### enabled

```
public abstract boolean enabled
```

:   Whether methods on this class/method are enabled.

    :   **Default:**: true

---


### groups

```
public abstract java.lang.String[] groups
```

:   The list of groups this class/method belongs to.

    :   **Default:**: {}

---


### dependsOnGroups

```
public abstract java.lang.String[] dependsOnGroups
```

:   The list of groups this method depends on. Every method
    member of one of these groups is guaranteed to have been
    invoked before this method. Furthermore, if any of these
    methods was not a SUCCESS, this test method will not be
    run and will be flagged as a SKIP.

    :   **Default:**: {}

---


### dependsOnMethods

```
public abstract java.lang.String[] dependsOnMethods
```

:   The list of methods this method depends on. There is no guarantee
    on the order on which the methods depended upon will be run, but you
    are guaranteed that all these methods will be run before the test method
    that contains this annotation is run. Furthermore, if any of these
    methods was not a SUCCESS, this test method will not be
    run and will be flagged as a SKIP.
    If some of these methods have been overloaded, all the overloaded
    versions will be run.

    :   **Default:**: {}

---


### alwaysRun

```
public abstract boolean alwaysRun
```

:   For before methods (beforeSuite, beforeTest, beforeTestClass and
    beforeTestMethod, but not beforeGroups):
    If set to true, this configuration method will be run
    regardless of what groups it belongs to.
      
    For after methods (afterSuite, afterClass, ...):
    If set to true, this configuration method will be run
    even if one or more methods invoked previously failed or
    was skipped.

    :   **Default:**: false

---


### inheritGroups

```
public abstract boolean inheritGroups
```

:   If true, this @Configuration method will belong to groups specified in the
    @Test annotation on the class (if any).

    :   **Default:**: true

---


### description

```
public abstract java.lang.String description
```

:   The description for this method. The string used will appear in the
    HTML report and also on standard output if verbose >= 2.

    :   **Default:**: ""

---


### timeOut

```
public abstract long timeOut
```

:   The maximum number of milliseconds this method should take.
    If it hasn't returned after this time, this method will fail and
    it will cause test methods depending on it to be skipped.

    :   **Default:**: 0L


---


|  |  |  |
| --- | --- | --- |
| **Overview** \| **Package** \| **Class** \| **Tree** \| **Deprecated** \| **Index** \| **Help** | |  |
| **PREV CLASS**   **NEXT CLASS** | **FRAMES**    **NO FRAMES**     **All Classes** |  |
| SUMMARY: REQUIRED \| OPTIONAL | DETAIL: ELEMENT |  |


---
